# Analysis on efficacy of compound dextran combined with atorvastatin calcium in patients with CVS caused by SAH based on TCD blood flow indexes

**DOI:** 10.12669/pjms.38.8.5504

**Published:** 2022

**Authors:** Yu Zhang, Feifan Ma, Ning Gan, Fei Su, Zhaoyan Song

**Affiliations:** 1 Yu Zhang, Department of Neurosurgery, Baoding No.1 Central Hospital, Baoding, 071000, Hebei, China; 2 Feifan Ma, Department of Cardiac Intensive Care, Baoding No.1 Central Hospital, Baoding, 071000, Hebei, China; 3 Ning Gan, Department of Neurosurgery, Baoding No.1 Central Hospital, Baoding, 071000, Hebei, China; 4 Fei Su, Department of Neurosurgery, Baoding No.1 Central Hospital, Baoding, 071000, Hebei, China; 5 Zhaoyan Song, Department of Neurosurgery, Baoding No.1 Central Hospital, Baoding, 071000, Hebei, China

**Keywords:** Blood flow indexes, Compound dextran, Atorvastatin calcium, Cerebral vasospasm caused by subarachnoid hemorrhage, Peroxidase 2, Endothelin-1

## Abstract

**Objectives:**

To analyze the effects of compound dextran combined with atorvastatin calcium on blood flow indexes, peroxidase 2 (Prx2) and endothelin-1 (ET-1) in patients with cerebral vasospasm (CVS) caused by subarachnoid hemorrhage (SAH).

**Methods:**

One hundred patients with CVS caused by SAH treated in Baoding No.1 Central Hospital from January 2019 to December 2020 were divided into observation group and control group. The control group was treated with atorvastatin calcium tablets, while the observation group was additionally treated with compound dextran. The hospital stays, Glasgow Outcome Scale (GOS), hemoglobin (Hb) and hematocrit (Hct) levels before and five days after treatment were recorded. The hemodynamic parameters of the middle cerebral artery (MCA) and the serum levels of Prx2 and ET-1 were detected.

**Results:**

After treatment, GOS and Hct levels in the observation group were both higher than those in the control group (*P* < 0.05). After treatment, the mean and peak velocities of the MCA in the observation group were significantly lower than those in the control group (*P* < 0.05). After treatment, the serum levels of Prx2 and ET-1 in the observation group were significantly lower compared with those in the control group (*P* < 0.05). However, no significant differences were found in the incidences of adverse reactions between the two groups (*P* > 0.05).

**Conclusions:**

Compound dextran combined with atorvastatin calcium can effectively enhance clinical efficacy, improve cerebral blood flow and reduce serum Prx2 and ET-1 levels in patients with CVS caused by SAH.

## INTRODUCTION

Subarachnoid hemorrhage (SAH) is one of the common neurosurgical disease in clinic and often divided into non-traumatic and traumatic, in which the non-traumatic is also known as spontaneous SAH.^1^ The common pathogeny of SAH includes arteriovenous malformation, aneurysm rupture, dural arteriovenous fistula, hypertensive cerebral hemorrhage, etc. It has been pointed out that SAH may often induce cerebral vasospasm (CVS), also known as delayed cerebral ischemia, which refers to vasospasm with cerebral infarction or ischemic symptoms. It is the most common complication of SAH at present, mainly manifested as decreased cerebral perfusion and vasospasm narrows, resulting in ischemic or delayed neurological damage.^2^ A survey shows that about 20%-40% SAH patients have vasospasm at different degrees, and CVS is also one of the important causes of stroke and even death.^3^ How to effectively improve CVS after SAH is one of the hotspots and difficulties in current clinical research. Dextran, the α-1,6-linked glucose polymer widely used in biology and medicine, promises new applications.^4^ Moreover, studies have shown that small-molecular-weight and low-molecular-weight dextran can significantly improve blood microcirculation, eliminate and prevent intravascular thrombosis and erythrocyte aggregation. They are commonly used in the clinical treatment of a variety of disseminated intravascular coagulation, microcirculation disorders, acute myocardial infarction, angina pectoris and other peripheral vascular diseases.^5^,^6^ However, at present, the application value of compound dextran in patients with CVS caused by SAH is still clinically controversial. Therefore, this study intends to select the patients with CVS caused by SAH treated in our hospital as subjects to explore the clinical efficacy of compound dextran, so as to provide data support for clinical promotion.

## METHODS

One hundred patients with CVS caused by SAH treated in Baoding No.1 Central Hospital from January 2019 to December 2020 who met the inclusion criteria were selected as subjects, and divided into observation group and control group using the random envelope method, with 50 patients in each group. The sample size required for each group was calculated by the formula


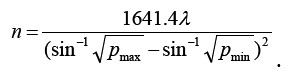


The general data showed no significant differences between the two groups (*P* > 0.05), suggesting reasonable grouping, as shown in Table I.

**Table-I:** Comparison of general data between the two groups.

Items	Observation group (n=50)	Control group (n=50)	P
Age (years)	59.93 ± 7.89	60.11 ± 8.93	0.46
Gender (male/female) (n)	21/29	22/28	0.37
Aneurysm (Yes/No) (n)	32/18	34/16	0.91
Surgery (Yes/No) (n)	28/22	26/24	0.62
Hypertension (Yes/No) (n)	19/31	18/32	0.88
Coronary heart disease (Yes/No) (n)	3/47	4/46	0.65
Diabetes (Yes/No) (n)	6/44	5/45	0.42
Antiplatelet drug therapy (Yes/No) (n)	3/47	2/48	0.56
Systolic blood pressure (mmHg)	140.92 ± 4.38	141.23 ± 7.89	0.77

### Ethical Approval:

The study was approved by the Institutional Ethics Committee of Baoding No.1 Central Hospital on May 27,2021(No.:2021[068]), and written informed consent was obtained from all participants.

### Inclusion Criteria:

- Patients with complete clinical data and treatment records.
- Patients diagnosed as spontaneous SAH by imaging examination.
- Patients diagnosed as CVS according to clinical symptoms and examination results.
- Patients signing the informed consent of this study.

### Exclusion Criteria:

- Hospital stay < 5 d;
- Aneurysm rerupture during hospitalization;
- Hunt-Hess grade V;
- Missing clinical or follow-up data.

All patients began to receive standardized treatment immediately after admission. The patients with clear surgical indications were treated with surgery according to their conditions, and intervened with basic therapy based on conventional anti-inflammation and electrocardiogram (ECG) monitoring. Maintain the patient’s blood volume and blood pressure. Both groups were given atorvastatin calcium tablets 10mg orally once a day. Nimodipine 30mg once a day was given intravenously according to the condition. The observation group was additionally given compound dextran 40 injection, 250ml, once a day, intravenous drip. The specification of compound dextran 40 injection is 250ml per bottle, containing 40 25g dextran, 50mg calcium chloride, 75mg potassium chloride, 1.5g sodium chloride, and 775mg sodium lactate. The treatment was continued for 5-12 days according to the recovery of the patients.

### Observation Indicators:

In this study, the length of hospital stays, Glasgow Outcome Scale (GOS) score, and hemoglobin (Hb) and hematocrit (Hct) levels before and five days after treatment were recorded in detail, and GOS score was used to evaluate the efficacy. In addition, the adverse reactions were recorded in detail. During the same period, the hemodynamic parameters of the middle cerebral artery (MCA) were detected in the temporal window with a 2-MHz probe using Multip X cranial Doppler blood flow analyzer (DWL, German). Moreover, fasting venous blood was collected from all the patients before treatment and five days after treatment, and serum was collected by centrifugation. Serum levels of peroxidase two (Prx2) and endothelin-1 (ET-1) were detected using enzyme-linked immunosorbent assay strictly in accordance with the instructions of the kit.

Statistical analysis was carried out using SPSS 20.0. The enumeration and measurement data were expressed as percentage and mean ± standard deviation, and their inter-group comparison was performed with the chi-square test and LSD-t test. *P* < 0.05 was considered as statistically significant.

## RESULTS

In this study, the results showed that the hospital stay of the observation group was significantly shorter than that of the control group (*P* < 0.05). Before treatment, GOS score, Hb and Hct levels had no significant differences between the two group (*P* > 0.05). After treatment, GOS score, Hb and Hct levels increased significantly in both groups (*P* < 0.05), and GOS score and Hct level in the observation group were higher than those in the control group (*P* < 0.05). However, no significant difference was found in Hb level between the two groups after treatment (*P* > 0.05), as seen in Table-II.

**Table-II:** Survey results of general treatment in patients.

Group	Hospital stays	GOS/score		Hb/g·L^-1^		Hct/%	

		Before treatment	After treatment	Before treatment	After treatment	Before treatment	After treatment
Observation group	14.12 ± 2.12	3.63 ± 0.44	4.84 ± 0.27	117.85 ± 13.22	126.84 ± 10.04	34.79 ± 1.32	38.59 ± 0.61
Control group	16.01 ± 3.11	3.59 ± 0.51	4.32 ± 0.32	119.03 ±1 0.74	122.19 ± 9.83	35.02 ± 1.15	36.84 ± 0.74
t	2.892	0.308	6.478	0.641	3.092	0.860	12.076
p	0.005	0.760	0.000	0.522	0.002	0.392	0.000

The results presented no significant differences in mean or peak velocities of MCA between the two groups before treatment (*P* > 0.05). After treatment, both mean and peak velocities of MCA in the two groups decreased significantly (*P* < 0.05), and the mean and peak velocities of MCA in the observation group were significantly lower than those in the control group (*P* < 0.05), as shown in Table-III.

**Table-III:** Hemodynamic parameters of MCA.

Group	Time	Mean velocity of MCA/cm/s	Peak velocity of MCA/cm/s
Observation group	Before treatment	138.59 ± 6.95	178.59 ± 24.11
	After treatment	50.92 ± 4.81	135.29 ± 14.36
Control group	Before treatment	140.24 ± 6.15	182.38 ± 20.94
	After treatment	58.91 ± 5.03	148.93 ± 10.99

Our results demonstrated no significant differences in serum Prx2 or ET-1 level between the two groups before treatment (*P* > 0.05). After treatment, serum Prx2 and ET-1 levels reduced significantly in both groups (*P* < 0.05), and those in the observation group were significantly lower compared with the control group (*P* < 0.05) (Table-IV).

**Table-IV:** Detection results of serum Prx2 and ET-1 levels in patients.

Group	Time	Prx2/ng/mL	ET-1/ng/mL
Observation group	Before treatment	2.84 ± 0.21	2.34 ± 0.51
	After treatment	0.98 ± 0.22	1.05 ± 0.19
Control group	Before treatment	2.79 ± 0.19	2.41 ± 0.48
	After treatment	1.43 ± 0.23	1.39 ± 0.23

### Adverse Reactions:

The results also revealed that in the observation group, there was one patient with urticaria, one patient with rashes and two patients with gastrointestinal symptoms such as vomiting and nausea. In the control group, one patient presented gastrointestinal symptoms such as vomiting and nausea, and one had mild liver function damage. The incidences of adverse reactions showed no significant differences between the two groups (*P* > 0.05).

## DISCUSSION

SAH is a common critical illness of the nervous system in clinic, and CVS is a common SAH-related nervous system complication. Vasospasm is a multifaceted and complex physiological and pathological process, involving many processes including automatic regulation and microcirculation. However, the exact mechanism of the occurrence and development of vasospasm has not been fully revealed.^7^-^9^ It has been shown that the risk of vasospasm is significantly correlated with the amount of SAH, and erythrocyte degradation in the subarachnoid space synchronizes with the onset of vasospasm.^10^,^11^ Thus, it is speculated that erythrocyte degradation products may be one of the inducing factors of vasospasm. In addition, some studies have pointed out that oxidized Hb, a degradation product of Hb, plays a major role in vasospasm. It can also stimulate the synthesis and release of arachidic acids, ET-1 and other vasoconstrictive substances, inhibit the synthesis of vasodilators and stimulate smooth muscle contraction, thereby aggravating vasospasm.^12^-^14^ Clinically, cerebral vasospasm after SAH is usually treated by maintaining blood volume and blood pressure, adding colloid fluid and intravenous dopamine drops when necessary. This method can increase the cerebral blood volume and improve the prognosis of patients, but its application in CVS treatment is still controversial. It has been pointed out that its application in the treatment of patients can easily lead to respiratory failure, pulmonary edema, renal insufficiency, heart failure and brain edema.^15^,^16^ Furthermore, studies have demonstrated that compared with hemodilution or hypervolemia therapy, the effect of induced hypertension therapy in improving cerebral blood flow is more accurate.^17^ Compound dextran contains not only dextran-coated colloids, but also supplementary crystal components including sodium chloride, sodium lactate, calcium chloride and potassium chloride. After intravenous drip, it can absorb extravascular water, improve plasma colloid osmotic pressure, maintain blood pressure and increase blood volume.^18^ The injection volume of dextran is closely related to plasma volume. It can depolymerize the aggregated platelets and erythrocytes, improve microcirculation, reduce blood viscosity and prevent thrombosis. Atorvastatin has been reported to alleviate early brain injury following SAH via reducing reactive oxygen species, antiapoptosis, regulated autophagy, and neuroinflammation.^19^

Prx2 is an important thiol-specific antioxidant protein, which exists in the form of homodimer in vivo. It is widely expressed in microglias of the brain tissue and plays an important role in antioxidation. A study has revealed that Prx2 can effectively remove hydrogen peroxide and avoid tissue and cell damage caused by oxidative stress.^20^ ET-1, an endogenous vasoactive substance synthesized and released by vascular endothelial cells, has a strong vasoconstrictive function and plays a very important role in the pathogenesis and development of multiple vascular diseases.^21^ Clinically, SAH is usually induced by rupture and hemorrhage of intracranial aneurysms. As a degradation product of Hb, oxidized Hb can stimulate myocardial cells or vascular endothelial cells to release ET-1, resulting in the increase of ET-1 level. Moreover, it can effectively reflect the state of myocardial cells and blood vessels by evaluating ET-1 level. When the expression level of ET-1 increases, it will bind to specific receptors, activating guanylate cyclases, opening calcium channel, increasing calcium ions in smooth muscle cells, and contracting vascular smooth muscle cells, which finally lead to the occurrence and development of CVS.

The results showed that after treatment, GOS score, Hb and Hct levels of the two groups increased significantly, and GOS score and Hct level of the observation group were higher than those of the control group. After treatment, the mean and peak velocities of MCA in the two groups decreased significantly, and those in the observation group were significantly lower than those in the control group. Further analysis of serum Prx2 and ET-1 levels revealed that the serum levels of Prx2 and ET-1 in the two groups reduced significantly after treatment, and the levels in the observation group were significantly lower compared with the control group. The survey results of adverse reactions demonstrated that there was one patient with urticaria, one patient with rashes and two patients with gastrointestinal symptoms such as vomiting and nausea in the observation group. In the control group, one patient presented gastrointestinal symptoms such as vomiting and nausea, and one had mild liver function damage. Earlier studies have suggested that dextran is safe and effective in preventing late cerebral vasospasm after subarachnoid hemorrhage.^22^ Compound dextran combined with atorvastatin calcium is safe in the treatment of CVS caused by SAH, and will not induce severe adverse reactions.

### Limitations of this study:

The number of subjects included in this study was limited, so the conclusions drawn may not be very convincing. In addition, there were few studies on compound dextran and CVS induced by SAH in previous studies, which proves the innovation of this study, but also makes the discussion of this study insufficient.

## CONCLUSION

In conclusion, compound dextran combined with atorvastatin calcium can effectively enhance clinical efficacy, improve cerebral blood flow and reduce serum Prx2 and ET-1 levels in patients with CVS caused by SAH. However, no long-term follow-up was performed in this study, which needs further study and analysis.

### Authors’ Contributions:

**YZ** & **FM:** Designed this study, prepared this manuscript, are responsible and accountable for the accuracy and integrity of the work.

**NG** & **FS:** Collected and analyzed clinical data.

**ZS:** Data analysis**,** significantly revised this manuscript.
